# Electrochemical Quantification of the Antioxidant Capacity of Medicinal Plants Using Biosensors

**DOI:** 10.3390/s140814423

**Published:** 2014-08-08

**Authors:** Erika Rodríguez-Sevilla, María-Teresa Ramírez-Silva, Mario Romero-Romo, Pedro Ibarra-Escutia, Manuel Palomar-Pardavé

**Affiliations:** 1 Departamento de Química, Universidad Autónoma Metropolitana-Iztapalapa, Área de Química Analítica, San Rafael Atlixco 186, Col. Vicentina, Del. Iztapalapa, México D.F., C.P. 09340, Mexico; E-Mail: chinita022001@yahoo.com.mx; 2 Departamento de Materiales, Universidad Autónoma Metropolitana-Azcapotzalco, Área Ingeniería de Materiales, Av. San Pablo 180, Col. Reynosa-Tamaulipas, Del. Azcapotzalco, México, D.F., C.P. 02200, Mexico; E-Mail: mmrr@correo.azc.uam.mx; 3 SEP-Instituto Tecnológico de Toluca. Departamento de Ingeniería Química y Bioquímica. Av, Tecnológico S/N. Fraccionamiento La Virgen, Metepec, Edo de México, C.P. 52149, Mexico; E-Mail: pedroibes@hotmail.com

**Keywords:** medicinal plants, antioxidant capacity, biosensors, tyrosinase, immobilization, screen-printed electrodes, Michaelis-Menten constant

## Abstract

The working area of a screen-printed electrode, SPE, was modified with the enzyme tyrosinase (Tyr) using different immobilization methods, namely entrapment with water-soluble polyvinyl alcohol (PVA), cross-linking using glutaraldehyde (GA), and cross-linking using GA and human serum albumin (HSA); the resulting electrodes were termed SPE/Tyr/PVA, SPE/Tyr/GA and SPE/Tyr/HSA/GA, respectively. These biosensors were characterized by means of amperometry and EIS techniques. From amperometric evaluations, the apparent Michaelis-Menten constant, *K_m_*′, of each biosensor was evaluated while the respective charge transfer resistance, Rct, was assessed from impedance measurements. It was found that the SPE/Tyr/GA had the smallest *K_m_*′ (57 ± 7) μM and Rct values. This electrode also displayed both the lowest detection and quantification limits for catechol quantification. Using the SPE/Tyr/GA, the Trolox Equivalent Antioxidant Capacity (TEAC) was determined from infusions prepared with “mirto” (*Salvia microphylla*), “hHierba dulce” (*Lippia dulcis*) and “salve real” (*Lippia alba*), medicinal plants commonly used in Mexico.

## 1. Introduction

Lately, enzymes have been used to manufacture biological sensors better known as biosensors [1,2], which are defined as compact analytical devices that incorporate a biological recognition element, like an acid, an enzyme, an antibody, a tissue, a cell, or a biomimetic, like molecular imprinted polymers or MIPs, aptamers, associated to a transduction system that performs signal processing as this is produced through interaction between the recognition element and the analyte.

The biosensor's detection principle is based on the specific interaction between the compound of interest and the recognition element. As result of such a bond there emerges a variation in one or more physicochemical properties, like pH, heat, mass or electron transfer, potential difference, variation of optical properties, and others, detected by the transducer. This system transforms the recognition element's response into an electronic signal directly linked to the presence of the analyte or proportional to its concentration in the sample under scrutiny [3–5]. The enzymes' immobilization plays a truly relevant role in biosensor manufacture, since it is used as recognition element, whereby the useful life, the sensitivity, the detection and the quantification limits of each sensor depend on the enzyme. There are several methods to effect immobilization [6,7], some of which occur through physical interactions such as entrapment, the inclusion in membranes or through microencapsulation and others through chemical interactions between the support and the enzyme like in ionic adsorption, covalent bonding and cross-linking, such that the following questions arise: what's the best immobilization method? What happens to all the general features of a given enzyme upon immobilization through any of the said methods? Or for a particular enzyme, which immobilization method likely leads to the design and fabrication of a robust biosensor?

It is recommended to perform a good selection of the immobilization method depending on two basic factors to be economized: time and resources. Above all, to obtain good reproducible results, the methods most used are physical adsorption, entrapment, cross-linking and covalent bonding.

Tyrosinase (Tyr), is an enzyme of the oxidases group comprising two copper atoms at meta position, oxy and desoxy states in its active sites (T3 sites), their function being to catalyze the *ortho*-hydroxylation of monophenols and oxidation of *o*-diphenols to *o*-quinones [8–10]; therefore, it can be immobilized through diverse methods with the aim of improving the biosensor's catalytic properties [11–14]. There are several mathematical models to evaluate the kinetic, catalytic or physicochemical properties of each enzyme, one of which is the Hill's model, based on Equation (1):

(1) $$R=R_{\max}\frac{{\left(\frac{\left[S\right]}{{\left[S\right]}_{0.5}}\right)}^{h}}{1+{\left(\frac{\left[S\right]}{{\left[S\right]}_{0.5}}\right)}^{h}}$$

where *R* is the biosensor's response; [S] is the substrate's concentration; *R_max_* is *R*'s limiting value when [S] → ∞; [S]_0.5_ is the media's saturation concentration, *i.e.*, when *R = R_max_ /2* and *h* is Hill's coefficient [15–20], which when *h* > 1 it implies cooperative kinetics, *h* = 1 describes Michaelian kinetics and *h* < 1 implies negative cooperativity between the enzyme and the substrate [21]. Whenever the Hill's coefficient = 1, Equation (1) becomes the Michaelis-Menten Equation (2), where [*S*]_0.5_ = Michaelis-Menten (*K_m_*) constant:

(2) $$R=R_{\max}\frac{\left[S\right]}{K_{m}+[S]}$$

In order to evaluate the features of an enzyme in solution the Michaelis-Menten model has been most used, where the reaction rates are measured as a function of the concentration of the substrate akin to the enzyme. In order to obtain the kinetic parameters like the *R_max_* and the *K_m_* the Lineweaver-Burk model, see Equation (3), also known as double reciprocal is used, which is the linearization of Equation (2):

(3) $$\frac{1}{R}=\frac{K_{m}}{R_{\max}}\frac{1}{\left[S\right]}+\frac{1}{R_{\max}}$$

Hence, this allows obtaining the *R*_max_ through the intercept while *K_m_* is associated to the slope of the straight line. If the Hill's coefficient differs from unit, then it is necessary to use a different model to that of Lineweaver-Burk. This model (see Equation (4)) results from the linearization of Hill's equation:

(4) $$\log\frac{R}{R_{\max}-R}=h\log[s]-h\log{\left[S\right]}_{0.5}$$

This system of equations (Equations (1)–(4)) allows evaluation of the same parameters, even when the enzyme is immobilized, in which case the mathematical model is adequately fitted, though the only change refers to notation of the Michaelis constant, that now receives the name of apparent Michaelis-Menten constant, *K_m_′* because the enzyme is not found in solution as in the original model. However, the use of these models (Equations (3) and (4)), generates kinetic constants with a large error, hence it is better to use constants, fitted by a non-linear regression method directly applied to Equation (1).

Either the Hill's or the Michaelis-Menten model is based on the reaction scheme (R1):

(R1) $$E+S\overset{k_{1}}{\underset{\underset{k_{-1}}{\leftarrow }}{\to }}ES\overset{k_{2}}{\to }E+P$$

where *E* represents the enzyme, *S* the substrate, *k_1_* is the enzyme-substrate's complex (*ES*) formation rate constant, *k_2_* is the product's (*P*) formation rate constant and *k_−1_* is the enzyme-substrate's complex decomposition rate constant. The apparent Michaelis-Menten (*K_m_′)* constant is defined as:

(5) $${K^{\prime }}_{m}=\frac{k_{-1}+k_{2}}{k_{1}}$$

which is inversely proportional to *k_1_*, therefore, if the value of the apparent Michaelis-Menten constant is large, it is due to the enzyme-substrate's complex (*ES*) formation rate constant, therefore, the product's reaction rate is small.

It is through the apparent Michaelis-Menten constant that descriptive information on the system can be obtained and used as a descriptive parameter of the sensor's robustness, although up to now it has either been used as another feature of the sensor without fitting the mathematical model to the experimental results, or without mentioning the type of kinetics described in the reaction [22–25].

Electrochemical impedance spectroscopy is a noninvasive, linear response electrochemical technique, which involves the perturbation of the system under study by a current or potential sinusoidal signal with variable frequency, where the transfer function obtained from the perturbation-response relationship is known as impedance. Today this technique has been used as a tool in various study fields such as batteries, fuel cells electrode kinetics, biosensors and biological processes, among others [26–28].

The construction of a robust biosensor involves obtaining analytically important parameters like life span, sensitivity, detection and quantification limits, each of which are found directly linked to the system's immobilization and therefore to the apparent Michaelis-Menten constant, since the more effective the enzymes' immobilization, then the greater the formation rate of the products. The use of EIS leads to a description of the sensor's surface and the charge transfer resistance for each immobilization, which points out to the enzyme's interaction with the substrate.

Recently, different biosensors have been reported in order to measure the antioxidant capacity of wine [29], fruit [30], infusions [31] and medicinal plant extracts [32]. The present research work shows that after mushroom tyrosinase is immobilized through the entrapment and cross-linking methods, under three different experimental conditions, the apparent Michaelis-Menten constant for all of them is used as a parameter to establish the system's kinetic behavior, the reaction product's formation rate and other kinetic, physicochemical and analytic parameters. Above all, it allows establishing the effect of the enzyme's active sites upon its immobilization for each of the methods described through the determination of *K_m_′* for tyrosinase's and catechol enzymatic oxidation. Such results are reinforced when characterizing each biosensor through EIS, also aided by SEM. Further, the determination of the Trolox Equivalent Antioxidant Capacity (TEAC), using the best method of immobilization in infusions of medicinal plants from Mexico such as mirto (*Salvia microphylla*), which is used popularly to relieve headache, drinking a cup of infusion before bed, hierba dulce (*Lippia dulcis*) used to relieve coughs as well as stomach aches, also drinking a cup of infusion before breakfast, and salve real (*Lippia alba*), which is a plant used to alleviate stomach ache and diarrhea, taking two or three cups of infusion per day.

## 2. Experimental Section

### 2.1. Reactants

The 34 U mg^−1^ mushroom tyrosinase (EC 232-653-4) was from Sigma (St. Louis, MO, USA), the water-soluble polyvinyl alcohol polymer, PVA-AWP, from Toyo Gosei Co., Ltd. (Tokyo, Japan), the 99% pyrocatechol from Fluka (St. Louis, MO, USA), the 25% glutaraldehyde (GA), at and the human serum albumin (HSA), were from Aldrich (St. Louis, MO, USA). For the buffer solution, 99.6% purity potassium phosphate dibasic (K_2_HPO_4_) (3252) and the 99.36% purity potassium phosphate monobasic (KH_2_PO_4_) were from Baker Analyzed (Center Valley, PA, USA), as well as the potassium chloride (KCl), the glacial acetic acid (CH_3_COOH), and the 99% purity sodium acetate (NaCH_3_COO). 2,2-Diphenyl-1-picryl-hydrazyl (•Dpph), and 6-hydroxy-2,5,7,8-tetrametyl choman-2-carboxylic acid (Trolox), and ethanol were from Aldrich. All solutions were prepared with deionized water type I.

### 2.2. Methods

#### 2.2.1. Spectrophotometric Characterization of Tyrosinase and Catechol in Solution

The spectrophotometric studies were carried out putting 145 μg·mL^−1^ mushroom tyrosinase and/or catechol 118 μM in 0.1 M acetate buffer at pH 4.50 ± 0.01 and at (30.0 ± 0.5) °C, using a Perkin Elmer Lambda 20 spectrophotometer (Waltham, MA, USA).

#### 2.2.2. Electrochemical Characterization of Tyrosinase and Catechol in Solution

An unmodified screen-printed electrode (SPE), having a 0.7 cm^2^ exposed surface area working electrode, was used for the amperometric determination of tyrosinase and catechol in solution, assessing the current as a function of the catechol's concentration at 5 μg·mL^−1^ mushroom tyrosinase. Six different methods were followed in order to characterize the catechol's using the immobilized tyrosinase biosensors, although for both characterizations a BAS LC-4C detector (West Lafayette, IN, USA) was used, imposing a −300 mV constant potential using a Ag/AgCl pseudoreference electrode [33], doing the determination in 0.1M acetate buffer at pH 4.50 ± 0.01 and at (30.0 ± 0.5) °C, with constant stirring.

### 2.3. Construction of the Biosensors

#### 2.3.1. Entrapment

Five μL of the mix 50% v/v 5 mg·mL^−1^ mushroom tyrosinase (Tyr) solution and water-soluble polyvinyl alcohol (PVA) are deposited over the screen-printed working electrode; subsequently, the electrodes are left to photocure for 3 h at 4 °C; this biosensor was labeled as SPE/Tyr/PVA [33].

#### 2.3.2. Cross-Linking with Albumin

Five mg·mL^−1^ of an enzymatic solution is prepared from mushroom tyrosinase in 5 mg·mL^−1^ has. This sort of albumin has been successfully used for biosensor fabrication [34], then a 50% v/v mix of the enzymatic solution with 2.5% GA was prepared and 5 μL of this solution were deposited over the working electrode and the membrane is left to polymerize at 4 °C for 3 h; this sensor was labeled as SPE/Tyr/HSA/GA. The optimization of the enzyme concentration, crosslinking agent and serum albumin quantities was carried out, in terms of the biosensor sensitivity, through the use of a 2^3^ experimental factorial design see Table S1 in the Supplementary Information of this paper.

#### 2.3.3. Cross-Linking without Albumin

A 50% v/v mix of 5 mg·mL^−1^ mushroom tyrosinase solution with 2.5% GA was prepared and then 5 μL of the mixture were deposited over the working electrode and the membrane's polymerization is allowed at 40 °C for 1 h; this sensor was referred to as SPE/Tyr/GA.

### 2.4. Determination of K_m_ and K_m_′

The Michaelis-Menten constant was obtained by fitting the Hill's and the Michaelis-Menten's models through non-linear regression with the aid of OriginLab 9. The apparent Michaelis-Menten's (*K_m_′*) constant was evaluated following the o-quinone's potentiostatic reduction, also called benzoquinone, formed enzymatically by mushroom tyrosinase for different catechol's concentrations

### 2.5. Sample Preparation

The medicinal plants were obtained from the area known as the hotland of the state of Guerrero, Mexico (coordinates: 17°37′N 99°57′W). The infusions were prepared as follows: for the mirto (*Salvia microphylla*) 4.8637 g of the plant including stem and leaves are placed in 50 mL H_2_O. For hierba dulce (*Lippia dulcis*) 2.4685 g of the plant including the stem, leaves and flowers, were placed in 50 mL H_2_O. For the salve real (*Lippia alba*) 7.8930 g of the plant including the roots, stem, leaves and flowers were placed in 50 ml H_2_O; all the solutions were prepared by infusing for 5 min in H_2_O at 100 °C, later the solution is decanted and cooled at room temperature.

### 2.6. Determination of the Antioxidant Capacity in Real Samples

To determine the antioxidant capacity using •Dpph spectrophotometric method [35] and Trolox as standard, the •Dpph radical absorbance was measured at 514 nm by varying the concentration of Trolox in the system. Calibration curves were constructed of the real sample for the determination of the Trolox Equivalent Antioxidant Capacity (TEAC); the results are reported in μg of Trolox per g of sample.

## 3. Results and Discussion

### 3.1. Evaluation of K_m_

**Figure 1.**
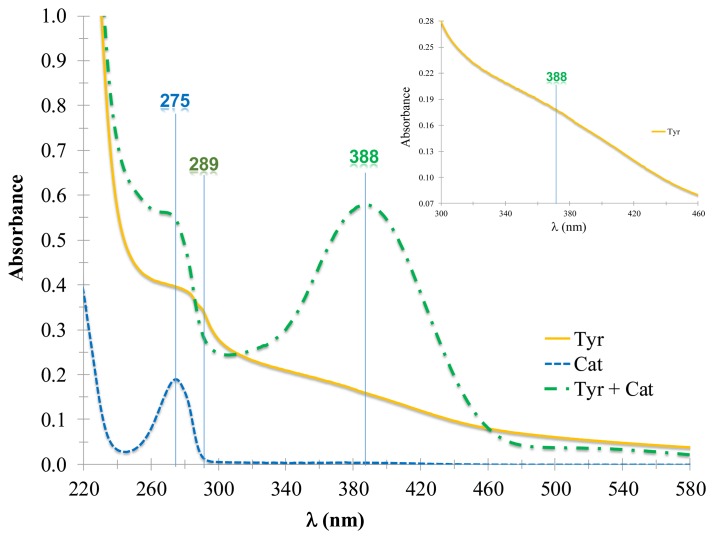
Absorption spectra of the mushroom tyrosinase (Tyr) 145 μg·mL^−1^ (**Solid line**), catechol (Cat) 118 μM (**Broken line**) and the reacting system Tyr + Cat; each spectrum was obtained in 0.1 M acetates' buffer at (30.0 ± 0.5) °C and at pH 4.50 ± 0.01. The inset shows a close-up of the Tyr absorption spectrum.

In order to carry out the spectrophotometric characterization of mushroom tyrosinase, the spectra of the enzyme and catechol were obtained, to be used as the enzyme's akin substrate, in 0.1 M acetate's buffer at pH 4.50 ± 0.01 and at (30.0 ± 0.5) °C. The tyrosinase (see Figure 1, solid line) shows an absorption band at 289 nm, and a shoulder at 388 nm, see the inset in Figure 1, whereas the catechol (see Figure 1, dotted line) showed an absorption band at 275 nm. The spectrum of the reacting system (Figure 1, dashed line) of tyrosinase (Tyr) with catechol (Cat), showed that the complex enzyme-substrate was formed, namely the tyrosinase-catechol (TyrCat), that dissociates afterwards to give the enzyme and the product: in this case benzoquinone (*o–Q*). There were two absorption bands observed, one at 275, which is attributed to the catechol that did not react in the system and another at 388 nm, attributed to benzoquinone formation, as indicated by reaction scheme R2:

(R2) $${\text{Tyr}}_{\left(aq\right)}+{\text{Cat}}_{\left(aq\right)}\Leftrightarrow {\text{TyrCat}}_{\left(aq\right)}\to {\text{Tyr}}_{\left(aq\right)}+o-Q_{\left(aq\right)}$$

**Figure 2.**
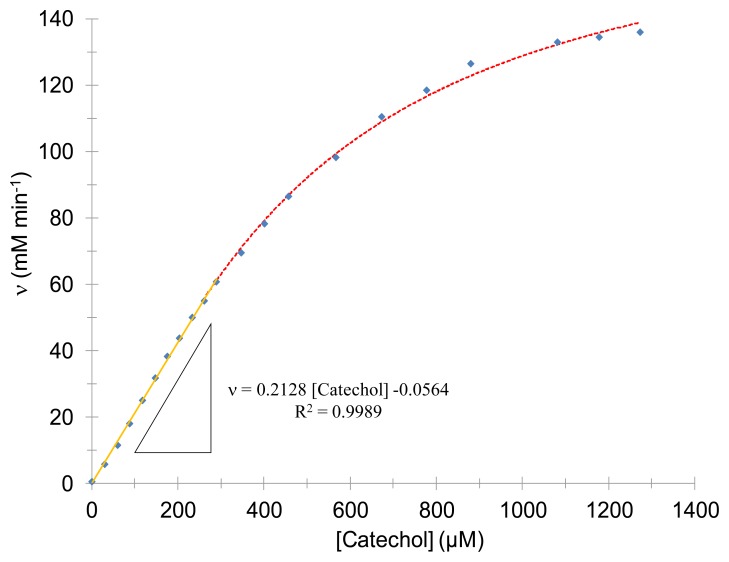
Enzymatic kinetics for catechol using mushroom tyrosinase in solution, in 0.1 M acetates' buffer at pH 4.50 ± 0.01 and at (30.0 ± 0.5) °C. Monitoring the enzymatic reaction rate as a function of the catechol's concentration; the dots are the experimental data while the line is the result of fitting the Hill's model Equation (1) to the data, using the values of *K_m_*= (0.46 ± 0.02) mM, v_max_= (174 ± 4) mM min^−1^ and *h* = 1.33 ± 0.04. The linear fit of the trace and its corresponding equation are also shown in the figure.

In order to obtain the kinetic parameters of the enzyme, it is adequate to monitor the absorbance at 388 nm, as a function of varying the catechol's concentrations. The reaction rate of the system formed by tyrosinase and catechol at (30.0 ± 0.5) °C in 0.1 M acetates' buffer at pH 4.50 ± 0.01 as a function of the catechol concentration [Catechol] is shown in Figure 2. In this case a linear interval given was observed given by: 0 μM ≤ [Catechol] ≤ 289 μM concentrations range with a linear regression coefficient of 0.9989. The corresponding kinetic parameters were also obtained through non-linear regression into the experimental data of the enzyme in solution *K_m_* = (460 ± 20) μM, v_max_ = (174 ± 4) μM·min^−1^ and *h* = 1.33 ± 0.04. It is important to state that the value for the Hill's coefficient obtained (*h*) refers to the cooperativity of the system, in agreement with Coperland [21], the tyrosinase shows a positive cooperativity with the substrate, thus the Hill's coefficient is greater than 1.

### 3.2. Evaluation of K_m_'

**Figure 3.**
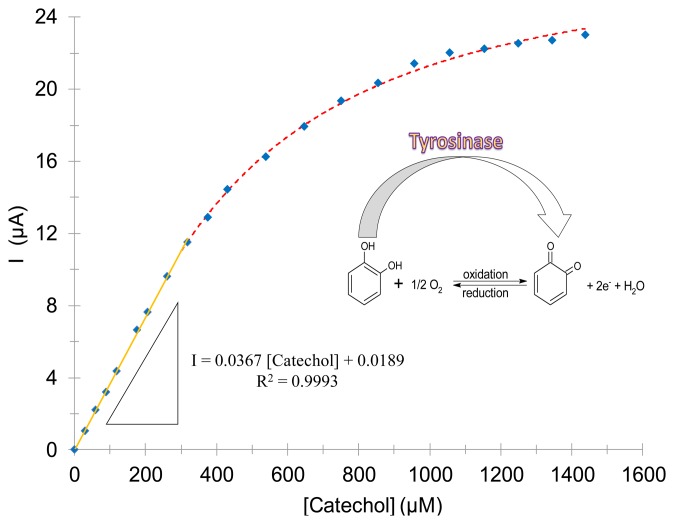
Enzymatic kinetics for catechol using mushroom tyrosinase in solution, in 0.1 M acetates' buffer at pH 4.50 ± 0.01 and at (30.0 ± 0.5) °C. Following the measured current at −300 mV imposed potential as a function of the catechol's concentration. The dots are experimental data and the line is the fitting of the Hill's model of Equation (1), the best fit parameters were *Km* = (0.40 ± 0.01) mM, *I_max_* = (27.5 ± 0.4) μA and *h* = 1.35 ± 0.03. The inset shows the reaction scheme of catechol and benzoquinone catalyzed by tyrosinase. The linear fit of the trace and its corresponding equation are also shown in the figure.

The amperometric evaluation of the tyrosinase in solution displayed a Michaelian-type kinetic behavior, (see Figure 3), where the current obtained due to the benzoquinone's reduction to catechol is monitored (inset Figure 3) at −300 mV imposed potential as a function of the catechol concentration, in 0.1 M acetate buffer at pH 4.50 ± 0.01 and at (30.0 ± 0.5) °C using an unmodified screen-printed electrode, SPE.

This electrode was used in order to study the kinetic behavior of the enzyme present in aqueous solution aiming to validate the estimation of the Michaelis-Menten constant using amperometry. Prior to determining the best potential to be imposed in the amperometric technique, a cyclic voltammetry study was carried in the system SPE/150 μM catechol, see Figure S1 in the Supplementary Information of this article. From this electrochemical study it is plain that at −300 mV the reduction of o–Q to catechol occurs with the maximum current; therefore, this potential was chosen to be applied during the amperometric study. In this case, the *K_m_′* = (404 ± 11) μM is similar to that obtained by means of UV-Vis spectroscopy, which allows neglecting a negative effect from the biosensor's material or from the pseudoreference used. However, the Michaelis-Menten constant was denoted as an apparent constant (*K_m_′*) because it was not obtained by means of the original model; *I_max_* = (27.5 ± 0.4) μA and *h* = 1.35 ± 0.03, which indicates that the system's cooperativity was not affected by the electrochemical technique used. The linear interval was: 0 μM ≤ [Catechol] ≤ 318 μM concentration range with a linear regression coefficient of 0.9992.

#### 3.2.1. Using the SPE/Tyr/PVA Biosensor

The immobilization of enzymes through the entrapment method consists of physically retaining the enzyme into the inner pores' surfaces in a porous solid matrix, generally built by photo-inter-cross-linked prepolymers or polyacrylamide-type polymers, collagen, alginate, carrageenan or polyurethane resins. Two relevant advantages of this method are that it requires small quantities of the enzyme to obtain active derivatives, which makes it is much simpler from the experimental point of view and secondly, that the enzyme does not suffer any structural alteration.

**Figure 4.**
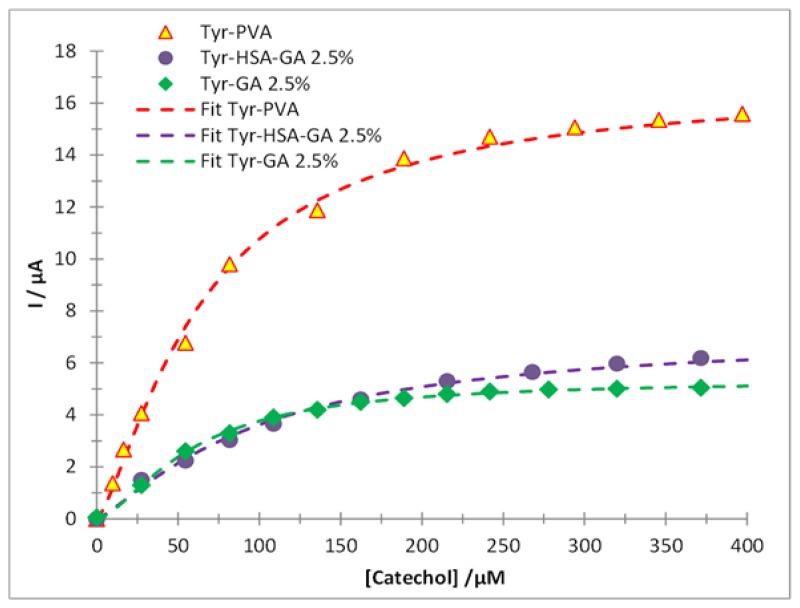
Comparison of the enzymatic kinetics for catechol (dots) recorded using the different tyrosinase biosensors: ( 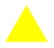) SPE/Tyr/PVA ( 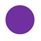) SPE/Tyr/HAS/GA and ( 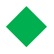) SPE/Tyr/GA in acetates' buffer 0.1M at pH 4.50 ± 0.01 a (30.0 ± 0.5) °C. Following the measured current, at −300 mV imposed potential, as a function of catechol's concentration. The lines correspond to the fitting of the Hill's model of Equation (1), the best fit values for *K_m_′, I_max_* and *h* are shown in Table 1.

Figure 4 shows the kinetic behavior of tyrosinase immobilized through entrapment; when the catechol concentration was varied in the system at pH 4.50 ± 0.01 imposed by means of 0.1 M acetate buffer at (30.0 ± 0.5) °C, in this case there was a linear interval of 0 μM ≤ [Catechol] ≤ 82 μM with R² = 0.9995, with a sensitivity of (120 ± 20) nA·μM^−1^, a LOD = (9.5 ± 0.4) μM and a LOQ = (31 ± 1) μM. In order to assess the immobilized enzyme's kinetic parameters the Hill's model is used as given in Equation (1); the type of kinetics for the enzyme and its substrate is cooperative with *h* = 1.35 ± 0.23. The kinetic parameters *I_max_* = (16.8 ± 0.2) μA and the *K_m_′* = (65 ± 2) μM indicate that the enzyme's catalytic activity has been improved respect to the enzyme's in solution.

#### 3.2.2. Using the SPE/Tyr/HSA/GA Biosensor

Cross-linking is another immobilization method that is carried out in two different manners: one is known as pure cross-linking, that has been widely used to stabilize many enzymes; the method uses bifunctional reactants (for example: dialdehides, diimine-ethers, diisocyanates, bisdiazonium salts, including, diamines, provided they are activated with carbodiimide), that give rise to intermolecular bonds among the enzyme's molecules. Cross-linking gives as result enzymes bearing irreversible intermolecular bonds, capable of resisting pH and temperature extreme conditions.

The kinetic behavior of the tyrosinase after immobilization with 2.5% glutaraldehyde (GA) as cross-linking agent is shown in Figure 4; the use of human serum albumin (HSA) involves functionalization of the enzyme active sites to turn them even more reactive. In this case the linear interval for the biosensor SPE/Tyr/HSA/GA is given as: 0 μM ≤ [Catechol] ≤ 109 μM with R² = 0.9950. The apparent Michaelis-Menten constant for the said sensor was (98 ± 6) μM, which either indicates that some of the enzyme's active sites are not adequately exposed or that that they have been blocked by the cross-linking agent. The sensor's analytical parameters are greater, LOD = (26 ± 1) μM, LOQ = (88 ± 32) μM and sensitivity (32 ± 1) nA·mM^−1^, compared to the immobilization through entrapment.

#### 3.2.3. Using the SPE/Tyr/GA Biosensor

Sensor fabrication aided by thermally assisted curing in the absence of HSA (SPE/Tyr/GA) process improved significantly the enzyme-substrate interaction (see Figure 4) thus increasing the sensor's enzymatic activity *K_m_′* = (57 ± 7) μM, with a linear interval given as: 0 μM ≤ [Catechol] ≤ 136 μM, a linear regression coefficient = 0.9998. In this case the enzymatic kinetics is cooperative *h* = 1.56 ± 0.03. The increase in enzyme activity affects the analytical parameters of the biosensor obtaining the lowest values of detection ((1.5 ± 0.6) μM) and quantification ((5.1 ± 1.7) μM) limits. This result proves that the apparent Michaelis-Menten constant is an indicative parameter of the sensor robustness, because lower values of *K_m_′* imply lower LOD and LOQ values. It is important to mention that the coefficient of variation (CV%) of this biosensor was 3.4%, estimated when the same biosensor was used to carry out 10 consecutive quantification measurements of a 50 μM Catechol solution, and 3.9% when 3 different biosensors were used.

A comparison of the immobilization methods for the mushroom tyrosinase is shown in Table 1; note that the immobilization through HSA-less cross-linking, with 2.5% glutaraldehyde at 40 °C is considered the best immobilization method because it improved the enzymatic activity, followed by the entrapment immobilization, and finally the HSA cross-linking with 2.5% glutaraldehyde, which exhibited the greatest loss of enzymatic activity.

### 3.3. Characterization of the Modified SPEs by Means of Electrochemical Impedance Spectroscopy, EIS and Scanning Electron Microscopy, SEM

**Figure 5.**
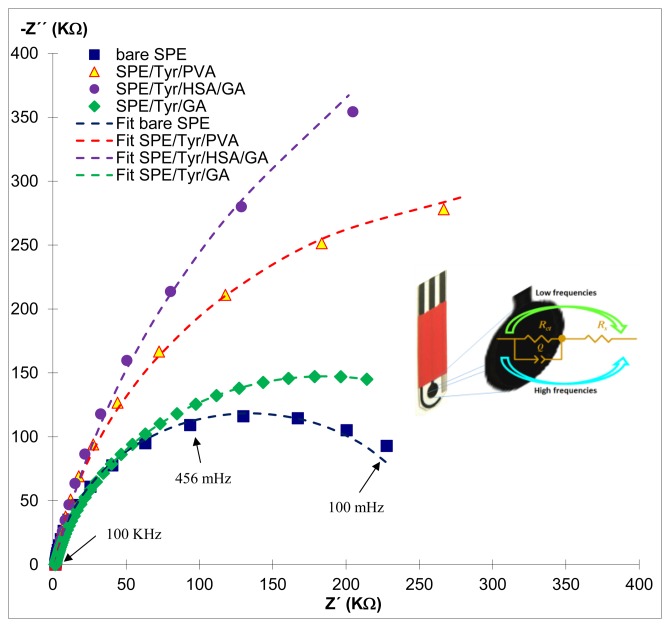
Comparison of the experimental Nyquist diagrams (dots) recorded using each biosensor immersed in 0.1 M phosphate's buffer at pH 7.00 ± 0.01, with 0.1 M KCl, 1 mM *Fe*(*CN*)_6_^3−/4−^ at (30.0 ± 0.5) °C, with the corresponding (broken line) obtained by fitting of the Randles' circuit, see the inset, to the experimental data using Zview software. With the best fit parameters the data shown in Table 2 were obtained.

It becomes possible to evaluate the porosity of the electrode using EIS; the effect of the membrane in charge transport as well as in the formation of the electrochemical double layer and for the evaluation of the limiting factors with respect to the charge transfer and the diffusion of the system in each biosensor. Figure 5 (dots) shows the Nyquist's diagram for the different biosensors built over the SPE electrodes, as well as the fitting (solid line) carried out through Zview, using the Randles circuit and the model (inset Figure 5), where *R_ct_* is the charge transfer resistance, *R_s_* is the solution resistance, *Q* is the electrochemical double layer's capacitance; the Nyquist's diagram shows the effect of adding each electrical component at the moment of fabricating the biosensor: it appeared that the capacitance has been considerably affected. The electrochemical impedance is calculated through Equation (6):

(6) $$Z=\frac{1}{C{\left(j\omega \right)}^{n}}$$

where *Z* is the impedance modulus, *C* is the double layer's capacitance, $j=\sqrt{-1}$, *ω* is the frequence: in agreement with Pajkossy [36] *n* is an indicative of rugosity or porosity inherent to the electrode that can adopt values from 0 to 1, where zero indicates that the electrode is not porous, while 1 means that the reverse is true.

Modifying of the surface of the working electrode of the SPE, makes that the *R_ct_* changes; the *R_ct_* for the biosensor SPE/Tyr/HSA/GA exceeds that of SPE/Tyr/PVA and is greater than for SPE/Tyr/GA (see Table 2), confirming once again that the best analytical parameters are obtained when the resistance to charge transfer and *K_m_′* are lower (see Table 1).

**Figure 6.**
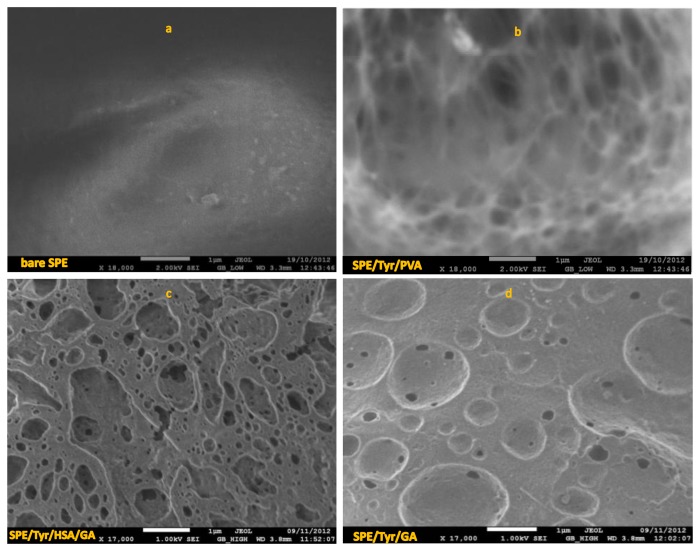
Results of the SEM morphology analysis of the bare SPE (**a**) and tyrosinase immobilized through: (**b**) entrapment, SPE/Tyr/PVA, (**c**) pure cross-linking with HSA, SPE/Tyr/HAS/GA and (**d**) cross-linking at (40.0 ± 0.5) °C, SPE/Tyr/GA.

When the surface of the SPE (see Figure 6a) is modified with Tyr/PVA (Figure 6b), the immobilization is carried out by forming a polymer network; hence it becomes more rugose (see the *n* values in Table 2), which affects directly the interaction between the enzyme and the substrate, thus the charge transfer resistance becomes larger (see the *R_ct_* values in Table 2). Modifying the sensor's surface with albumin makes it becomes more resistive, *R_ct_* = 1359 KΩ, in spite of the porous surface (see Figure 6c), which had a direct impact on the biosensor's analytic parameters: this being the reason for obtaining highest LOD and LOQ compared to the previous case, while the interaction between the enzyme and the substrate becomes favored (see Table 1). Finally when the surface of the SPE is modified with GA, polymerized at 40 °C, the membrane loses porosity, however, it is uniform porosity (Figure 6d); a GA's relative proportion of 2.5%, led to an improved enzyme's-substrate interaction, thus rendering biosensors with a smaller charge transfer resistance, namely 367 KΩ, providing hence robust sensors.

The Michaelis–Menten's constants values obtained are smaller than those reported in the literature for immobilization of tyrosinase immobilized through the same methods, including those that used nanoparticles [37–39], this indicates that the enzyme activity increases. Manufacturing these biosensors is simple and inexpensive because it does not use nanoparticles, Au electrodes, nanotubes, and so on. For example, the manufacture of robust biosensor prepared with glutaraldehyde is 80% cheaper compared to the other biosensors.

So far our results have shown that SPE/Tyr/GA displays the best attributes for a biosensor, namely: The smallest *K_m_′* and *R_ct_* values and the lowest detection and quantification limits for catechol quantification. Accordingly, this can be explained because the polymeric network formed by GA promotes two sorts of sites, one where the interaction between the enzyme and the substrate is favored, see reaction R2, thus giving the lowest *K_m_′* values, and a second one where the electrochemical reduction of the enzymatic reaction product takes place easily, thereby lowering the *R_ct_* value. The synergy between these two kinds of sites provides the SPE/Tyr/GA biosensor with the best analytical performance.

### 3.4. Quantification of the Trolox Equivalent Antioxidant Capacity

The Trolox Equivalent Antioxidant Capacity (TEAC) of real medicinal plant samples commonly used in the Mexican herbalist, namely: “mirto” (*Salvia microphylla*), “hierba dulce” (*Lippia dulcis*) and “salve real” (*Lippia alba*) was estimated using two different methods: spectrophotometric with DPPH and using the biosensor SPE/Tyr/GA. Table 3 shows that using the spectrophotometric method with DPPH led to the following trend for the TEAC values: *Salvia microphylla* > *Lippia dulcis* > *Lippia alba*. When using the biosensor the same trend was observed (see Table 3). It is important to mention that these plants exhibit TEAC values five times larger compared to other medicinal plant extracts [32]. This shows that the sensor is applicable to the determination of antioxidant capacity. Notwithstanding, it is relevant to note that for each sample the TEAC values estimated from DPPH method and using the biosensor are different. Diverse methodological approaches will provide information on different phsyiological aspects, see for instance the work of Kintzios *et al.* [40]. In the presence case the quantification of TEAC using the biosensor SPE/Tyr/GA is mainly given by the contribution of monophenolic compound.

## 4. Conclusions

The immobilization of mushroom tyrosinase through the cross-linking method, with 2.5% glutaraldehyde at 40 °C is considered the best because it improved the enzymatic activity, generated robust biosensors having better LOD, LOQ and sensitivity. Then it followed the immobilization through entrapment; one but last, it was the pure cross-linking method using HSA to functionalize the tyrosinse's active sites. The last was the cross-linking albuminless method with 2.5% GA, which displayed the largest loss of enzymatic activity.

The use of EIS permitted to evaluate the effect of the double layer for each system as well as the porosity of the sensor, which exhibited an interaction level between the enzyme and the substrate, thus showing that immobilization through cross-linking with GA at 40 °C generated rugose sensors that displayed good cooperativity of the system and rendering robust, inexpensive, easy to fabricate, reusable biosensors. The application in real samples, indicates that the proposed biosensor in this work is useful for determining TEAC and comparable with well-established methods.

## Figures and Tables

**Table 1.** Analytic parameters of the tyrosinase immobilized through entrapment, cross-linking and covalent bonding over screen-printed-type sensors.

**Method**	***K_m_′* (μM)**	**Sensitivity (nA·μM^−1^)**	**LOD/μM**	**LOQ/μM**	**Linear Range (μM)**	**I_max_ (μA)**	***h***	Useful Life Span *(Days)
Entrapment (SPE/Tyr/PVA)	65 ± 2	120 ± 2	9.5 ± 0.4	31 ± 1	0 ≤ [Catechol] ≤ 82	16.80 ± 0.23	1.35 ± 0.06	> 360
Cross-Linking (SPE/Tyr/GA)	57 ± 7	26 ± 4	1.5 ± 0.6	5.1 ± 1.7	0 ≤ [ Catechol] ≤ 136	5.35 ± 0.02	1.56 ± 0.03	> 165
Cross-Linking (SPE/Tyr/HSA/GA)	98 ± 6	32 ± 10	26 ± 1	88 ± 32	0 ≤ [ Catechol] ≤ 109	7.2 ± 0.2	1.21 ± 0.08	< 3

* The biosensor was stored at room temperature conditions.

**Table 2.** Characterization by means of EIS of the bare SPE and the biosensors.

**Biosensor**	***R_s_* (KΩ)**	***R_ct_* (KΩ)**	***C* (μF)**	***n***
SPE	1.67	265	0.007	0.92
SPE/Tyr/GA	1.62	368	0.430	0.87
SPE/Tyr/PVA	1.62	680	0.740	0.91
SPE/Tyr/HSA/GA	1.70	1359	0.552	0.88

**Table 3.** Quantification of the trolox equivalent antioxidant capacity of different real samples using the spectrophotometric method and the SPE-Tyr-GA 2.5% biosensor.

**Sample**	**TEAC _•Dpph_ μg of Trolox/mL**	**TEAC _SPE/Tyr/GA_ μg of Trolox/mL**
“Mirto” (*Salvia microphylla*)	620 ± 38	31 ± 1
“Hierba dulce” (*Lippia dulcis*)	519 ± 23	4.9 ± 0.2
“Salve Real” (*Lippia alba*)	459 ± 16	0.82 ± 0.03
